# Is the Serum Vitamin D Level at the Time of Hospital-Acquired Acute Kidney Injury Diagnosis Associated with Prognosis?

**DOI:** 10.1371/journal.pone.0064964

**Published:** 2013-05-22

**Authors:** Lingyun Lai, Jing Qian, Yanjiao Yang, Qionghong Xie, Huaizhou You, Ying Zhou, Shuai Ma, Chuanming Hao, Yong Gu, Feng Ding

**Affiliations:** Division of Nephrology, Huashan Hospital, Fudan University, Shanghai, China; University of Sao Paulo Medical School, Brazil

## Abstract

**Background:**

Low circulating vitamin D levels have been suggested to potentially contribute to acute complications in critically ill patients. However, in patients with acute kidney injury (AKI), whether vitamin D deficiency occurs and is a potential contributor to worse early outcomes at the time of AKI diagnosis remains unclear.

**Methodology/Principal Findings:**

Two hundred patients with AKI were enrolled in our study. Healthy subjects and critically ill patients without AKI served as controls. Serum vitamin D concentrations were measured in the three groups. The patients with AKI were followed up for 90 days and grouped according to median serum vitamin D concentrations. In addition, vitamin D receptor polymorphisms (*Bsm*I and *Fok*I) were measured in these patients; they were also followed up for 90 days and grouped according to vitamin D receptor gene mutations. Low serum 1,25-dihydroxyvitamin D levels (59.56±53.00 pmol/L) were detected in patients with AKI and decreased with increasing severity of AKI. There were no significant findings with respect to 25-hydroxyvitamin D. The 90-day survival curves of individuals with high vitamin D concentrations showed no significant differences compared with the curves of individuals with low concentrations. The survival curves of patients with *BB/Bb* or *FF/Ff* genotypes also showed no significant differences compared with patients with *bb* or *ff* genotypes. In Cox regression analysis, the vitamin D status in patients with AKI was not an independent prognostic factor as adjusted by age, sex, Sequential Organ Failure Assessment score, or vitamin D receptor polymorphisms.

**Conclusions/Significance:**

Patients with AKI manifested a marked decrease in the 1,25-dihydroxyvitamin D level at the time of AKI diagnosis, and the degree of 1,25-dihydroxyvitamin D deficiency increased with the severity of AKI. No association between the serum vitamin D level at the time of AKI diagnosis and 90-day all-cause mortality was found in patients with AKI.

## Introduction

Vitamin D deficiency is very prevalent in the adult population worldwide [1]–[3] and has been demonstrated to strongly correlate with long-term overall mortality in the general population [4]–[6]. Vitamin D has pleiotropic effects on immunity, endothelial and mucosal functions, and glucose and calcium metabolism [7]. These effects may account for the association between its deficiency and the increased mortality and morbidity associated with a variety of chronic illnesses such as coronary disease, tuberculosis, malignant tumors, and chronic renal disease.

Serious deficiency of 25-hydroxyvitamin D in critically ill patients and its association with increased mortality has been a focus of recent studies [8]–[13]. An observational study by Lee et al. [8] first indicated that the predicted mortality rates in critically ill patients with sufficient, insufficient, and deficient levels of 25-hydroxyvitamin D were 16%, 35%, and 45%, respectively. Braun et al. [12], [13] also showed that vitamin D deficiency prior to hospital admission or at the time of critical care is independently associated with increased morbidity and mortality in patients with critical illness. Vitamin D dysfunction might also contribute to common acute complications such as sepsis, organ failure, and systemic inflammatory response syndrome, leading to worse outcomes.

Despite this knowledge, the potential role of vitamin D system dysfunction has rarely been considered in patients with acute kidney injury (AKI). The 1α-hydroxylase responsible for the formation of 1,25-dihydroxyvitamin D from 25-hydroxyvitamin D is mainly located in the inner mitochondrial membrane of the renal proximal tubule epithelium [7], which is easily injured in AKI. This implies that circulating vitamin D deficiency, especially 1,25-dihydroxyvitamin D deficiency, is much more severe in patients with AKI. Whether such vitamin D deficiency caused by AKI is associated with increased overall mortality remains unknown. Moreover, vitamin D receptor (VDR) polymorphisms reportedly influence the expression and nuclear activation of the VDR, which has been considered to associate with different diseases such as malignant tumors and diabetes [14]. Finally, whether VDR polymorphisms are linked to the excess all-cause mortality risk in patients with AKI is unknown.

Accordingly, the aim of our study was to determine the vitamin D status, including the 25-hydroxyvitamin D and 1,25-dihydroxyvitamin D levels, at the time of AKI diagnosis and investigate the possible association between the low vitamin D status caused by AKI and the 90-day overall mortality rate.

## Materials and Methods

### Selection of Participants

#### Patients with AKI

We prospectively evaluated a consecutive cohort of 200 adult patients with hospital-acquired AKI from February 2009 to December 2009 at Huashan Hospital, a tertiary hospital attached to Fudan University with 30 wards and 1500 beds in Shanghai, China. Eligible patients were ≥18 years old and diagnosed with AKI during hospitalization. Exclusion criteria included 1) confirmed and/or suspected acute glomerulonephritis, acute interstitial nephritis, renal vasculitis, or postrenal etiology of AKI; 2) diagnosis of metastatic tumors; 3) admission with AKI; 4) unknown premorbid creatinine level; 5) enrollment in other studies; 6) pregnancy; and 7) use of medications containing vitamin D or calcium. All patients were followed up for 90 days. The primary outcome was all-cause mortality.

Baseline demographic and biochemical characteristics of patients with AKI are shown in Table 1.

**Table 1:** Baseline demographic and clinical data of patients at the time of acute kidney injury diagnosis stratified by RIFLE stages.

	No. (%)				
Characteristic	Total	Risk	Injury	Failure	*p* value
	(n = 200)	(n = 91)	(n = 50)	(n = 59)	
Age (yr), mean (s.d.)	63.7±18.8	66.7±16.9	67.5±20.2	55.7±18.1	<0.001
Gender(%female)	53(26.5)	20(22.0)	16(32.0)	17(28.8)	0.388
Baseline Scr (mg/dL)	0.7(0.5, 0.88)	0.74±0.26	0.70±0.28	0.65(0.46, 0.88)	0.189
Scr when AKI diagnosed (mg/dL)	1.46(1.18, 2.10)	1.27±0.40	1.59±0.52	3.82±2.63	<0.001
Baseline eGFR (ml/min/1.73 m^2^)	89.6(68.3, 110.0)	80.4(59.2, 110.3)	86.6(68.4, 104.4)	90.7(79.3, 126.5)	0.250
Comorbid conditions					
Hypertension (%)	86(45.0)	44(50.6)	19(39.6)	23(41.1)	0.366
CVD (%)	25(13.1)	15(17.2)	8(16.7)	2(3.6)	0.042
DM (%)	36(18.8)	15(17.2)	11(22.9)	10(17.9)	0.704
Chronic hepatic disease (%)	10(5.2)	7(8.0)	1(2.1)	2(3.6)	0.265
Malignant tumor (%)	17(8.9)	5(5.7)	5(10.4)	7(12.5)	0.350
CKD (%)	16(8.4)	8(9.2)	3(6.3)	5(8.9)	0.827
Operation (%)	41(20.6)	27(29.7)	11(22.0)	3(5.2)	0.001
Sepsis (%)	86(43.2)	36(39.6)	24(48.0)	26(44.8)	0.600
Mechanical ventilation (%)	62(31.0)	27(29.7)	14(28.0)	21(35.6)	0.648
MAP (mmHg)	88.0±17.9	89.3±14.4	86.0±15.3	87.6±23.9	0.571
WBC (×10^9^)	11.6(8.0, 16.6)	12.5±8.3	13.2±6.4	12.2±6.0	0.743
Neutrophilicgranulocyte (%)	82.8(76.6, 87.1)	81.4(74.4, 88.2)	85.1(77.5, 87.3)	82.8±7.9	0.250
Hemoglobin(g/L)	111.2±24.4	112.5±22.3	114.9±24.5	105.9±26.7	0.594
ALT (U/L)	32.5(20.0, 65.3)	29.0(19.5, 60.0)	34.0(20.0, 75.8)	34.0(19.0, 89.0)	0.781
AST (U/L)	41.0(25.0, 97.3)	37.0(25.0, 91.5)	47.0(27.8, 111.0)	42.0(21.0, 119.0)	0.450
Serum albumin (g/dL)	3.25±0.68	3.34±0.64	3.39±0.71	2.99±0.65	0.002
Serum total calcium (mmol/L)	2.06±0.25	2.09±0.21	2.11±0.27	1.99±0.28	0.030
Serum phosphate (mmol/L)	1.2(0.9, 1.6)	1.1±0.5	1.3±0.7	1.6±0.7	0.001
Cholesterol (mmol/L)	3.7±1.5	3.6±1.4	3.7±1.4	3.8±1.7	0.791
CRP (mg/L)	65.6(18.6, 119.0)	38.9(13.5, 111.8)	84.0±66.7	90.8±64.8	0.045
BMI (kg/m^2^)	22.26±3.57	22.35±3.63	21.70±3.46	22.60±3.59	0.426
SOFA	6.0(4.0, 11.0)	5.0(2.0, 8.0)	7.9±4.9	8.7±4.5	0.001
APACHE II	16.0(12.0, 26.0)	14.0(10.0, 21.8)	19.0±8.1	21.7±9.4	0.001
SAPS	45.6±19.0	41.1±16.9	47.7±17.4	50.7±21.9	0.007
SGA	2.0(1.0, 2.0)	2.0(1.0, 2.0)	2.0(2.0, 2.0)	2.0(1.0, 3.0)	0.767

**Note:** Data were obtained at the time of AKI diagnosis unless otherwise noted. eGFR was evaluated by the Modification of Diet in Renal Disease formula.

**Abbreviations:** RIFLE, Risk, Injury, Failure, Loss, and End-stage kidney disease; eGFR, estimated glomerular filtration rate; CVD, cardiovascular disease; DM, diabetes mellitus; CKD, chronic kidney disease; COPD, chronic obstructive pulmonary disease; Scr, serum creatinine; AKI, acute kidney disease; MAP, mean arterial pressure; WBC, white blood cells; ALT, alanine aminotransferase; AST, aspartate aminotransferase; CRP, C-reactive protein; BMI, body mass index; SOFA, Sequential Organ Failure Assessment; APACHE II, Acute Physiology and Chronic Health Evaluation II; SAPS, Simplified Acute Physiologic Score; SGA, Subjective Global Assessment.

#### Critically Ill Patients without AKI

Thirteen critically ill patients without AKI served as control subjects. All were inpatients of Huashan Hospital during the same period. The absence of AKI was determined by the serum creatinine level. All patients were matched according to age, gender, and Sequential Organ Failure Assessment (SOFA) score [15].

#### Healthy Subjects

A group of 17 age- and gender-matched healthy subjects were used for comparison. Healthy subjects were randomly obtained from among healthy patients of the health check-up center of Huashan Hospital during the same period.

None of the participants were taking drugs known to interfere with vitamin D levels during the 90-day follow-up, including vitamin D and antiepileptic drugs. Patients with AKI and critically ill patients without AKI remained under the care of the hospital unit to which they were admitted. The study investigators did not participate in the patients’ medical care unless invited. The study was approved by the ethics committee of Huashan Hospital, Fudan University (approval number: 2009-097). All patients gave written informed consent, and the Declaration of Helsinki was adhered to.

### Study Definitions

AKI was determined using the Risk, Injury, Failure, Loss, and End-stage kidney (RIFLE) classification criteria [16]. According to the RIFLE classification criteria, patients were diagnosed and their disease severity was classified based on changes in the serum creatinine level within 1 week. Patients’ disease was classified as stage Risk if their serum creatinine level was 1.5 times the baseline creatinine level, stage Injury if their serum creatinine level was twice the baseline level, and stage Failure if their serum creatinine level was three times the baseline level. The baseline serum creatinine level was defined by the lowest serum creatinine level within 1 week before diagnosis of AKI. According to consensus guidelines, sepsis syndrome was considered to be present in patients in whom infection was accompanied by at least two systemic inflammatory response syndrome criteria. Infection was diagnosed according to usual clinical, laboratory, and microbiological parameters. Patients with operations were defined as those who had undergone a surgical operation within 1 week before diagnosis of AKI.

### Blood Sampling

Blood samples were obtained from patients with AKI within 24 hours after AKI was first diagnosed. Blood samples were obtained from critically ill patients without AKI within 48 hours after admission. Samples were obtained from healthy subjects at the time of enrollment. Blood for serum measurements was drawn into BD Vacutainer serum-separating tubes (Becton, Dickinson and Company, Franklin Lakes, NJ) that contained a clot activator. For molecular genetic studies, blood was drawn into Vacutainer tubes containing ethylenediaminetetraacetic acid (EDTA) as an anticoagulant. Tubes were kept at room temperature and centrifuged within 1 hour of the blood draw. All blood samples were stored at –80°C until analysis.

### Clinical Evaluation

Baseline demographics were recorded, including age, gender, and comorbidities such as hypertension, diabetes mellitus, cardiovascular disease, chronic hepatic disease, chronic kidney disease, chronic obstructive pulmonary disease, and malignant tumors. The following data were also recorded upon patient enrollment: the possible cause of AKI, the presence of sepsis, and the need for mechanical ventilation. We further assessed the SOFA score, Acute Physiology and Chronic Health Evaluation (APACHE) II score [17], Simplified Acute Physiologic Score (SAPS), and Subjective Global Assessment (SGA) [18].

### Laboratory Procedures

The white blood cell, neutrophilic granulocyte, and hemoglobin concentrations were measured by an automated hematology analyzer (Sysmex XE-2100). The serum levels of creatinine, alanine aminotransferase, aspartate aminotransferase, albumin, calcium, and phosphate were determined by a biochemistry autoanalyzer (Hitachi 7600-020b).

The serum 25-hydroxyvitamin D level was determined by enzyme-linked immunosorbent assay (ELISA) with 25-hydroxyvitamin D enzyme immunoassay kits from Immunodiagnostic Systems Limited (United Kingdom). The sensitivity was 5 nmol/L. The intra-assay coefficient of variation was 5.3%, 5.6%, and 6.7% at a mean level of 39.0, 67.2, and 165 nmol/L, respectively. The inter-assay coefficient of variation was 4.6%, 6.4%, and 8.7% at a mean level of 40.3, 72.0, and 132 nmol/L, respectively. The cross-reactivity ratio with 25-hydroxyvitamin D_3_, 25-hydroxyvitamin D_2_, 24,25-dihydroxyvitamin D_3_, and cholecalciferol was 100%, 75%, ≥100%, and <0.01%, respectively.

The serum 1,25-dihydroxyvitamin D level was measured by 1,25-dihydroxyvitamin D enzyme immunoassay kits from Immunodiagnostic Systems Limited. The sensitivity was 6 pmol/L. At a mean level of 19.0, 53.2, and 152 nmol/L, the intra-assay coefficient of variation was 10.7%, 10.5%, and 9.3% and the inter-assay coefficient of variation was 19.7%, 17.1%, and 17.6%, respectively. The cross-reactivity ratio with 1,25-dihydroxyvitamin D_3_, 1,25-dihydroxyvitamin D_2_, 24,25-dihydroxyvitamin D_3_, and 25-hydroxyvitamin D_3_ was 100%, 39%, 0.056%, and 0.009%, respectively.

For the genotype analysis, genomic DNA was extracted from whole blood by the standard phenol-chloroform method. Details of the studied single nucleotide polymorphisms, polymerase chain reaction (PCR) primers, PCR condition, restriction fragment length polymorphism (RFLP) condition, and lengths of PCR products and RFLP products are shown in Table 2. The PCR products were digested overnight by corresponding restriction enzymes (New England Biolabs, Ipswich, MA), and the RFLP products were run on 2% agarose gel (Bio-Rad) and stained with ethidium bromide for visualization under ultraviolet light. The information regarding the studied markers for the VDR gene is shown in Table 2.

**Table 2:** Data on studied markers for the VDR gene.

SNPs	SNP reference number	Location/Base change	Forward primer /Reverse primer (Reference)	PCR condition	PCR fragment size (bp)	Restrictionenzyme, incubation temperature	RFLP fragments size (bp)
*Fok*I	rs10735810	Exon2	F: GGCAACCTGAAGGGAGACGTA	(40 cycles)	265	*Fok*I, 24°C	169, 96
		(C/T)	R: CTCTTTGGACCTCATCACCGAC	95°C 30s, 65°C 30s, 72°C 60s			
*Bsm*I	rs1544410	Intron8	F: AGCTGGCCCTGGCACTGACTCTGCTCT	(40 cycles)	461	*Bsm*I, 24°C	258, 203
		(A/G)	R: ATGGAAACACCTTGCTTCTTCTCCCTC	95°C 30s, 65°C 30s, 72°C 60s			

**Note:** Genotypes were expressed in RFLP nomenclature: uppercase letters denote the absence of a restriction site, while lowercase letters indicate the presence of a restriction site. In other words, VDR genotypes of each subject were identified according to the digestion pattern, and alleles were identified according to the presence (*f* or *b*) or absence (*F* or *B*) of the *Fok*I and *Bsm*I sites, respectively. The *F* and *B* alleles correspond to the C and A nucleotides, respectively.

**Abbreviations:** VDR, vitamin D receptor; SNPs, single nucleotide polymorphisms; PCR, polymerase chain reaction; RFLP, restriction fragment length polymorphism.

### Statistical Analyses

Normally distributed variables are expressed as mean ± standard deviation (SD) and were compared using one-way analysis of variance (ANOVA) or the *t* test. Non-normally distributed variables are expressed as medians with interquartile range and were compared using the rank sum test. Categorical variables are expressed as percentages and were compared using Pearson’s chi-square test or Fisher’s exact test. Correlations among continuous data were performed using Pearson’s correlation coefficients. Overall survival at 90 days among the groups was evaluated using Kaplan-Meier analysis, and differences among them were tested using the log-rank test. Cox proportional hazards regression was used to identify independent predictors of mortality in patients with AKI. Covariates including age, gender, SOFA score, and VDR polymorphisms were used for stepwise adjustment. The terminal event was death, and patients lost to follow-up were censored at their last observation. All tests were two-tailed, and statistical significance was defined as *p*<0.05. The SPSS statistical software program (version 15.0, SPSS Inc., Chicago, IL) was used for all analyses.

## Results

### Demographic and Clinical Characteristics of the AKI Study Cohort

Of the 200 patients (147 men and 53 women) with a mean age of 63.66±18.76 years enrolled in our study who met the criteria for AKI, 91 (45.5%), 50 (25%), and 59 (29.5%) reached the RIFLE stages Risk, Injury, and Failure, respectively, during hospitalization. Table 1 shows the demographic and clinical characteristics stratified by the RIFLE stage of AKI. The predominant comorbid conditions of the study cohort were as follows: hypertension in 86 patients (45.0%), cardiovascular disease in 25 (13.1%), diabetes mellitus in 36 (18.8%), chronic hepatic disease in 10 (5.2%), nonmetastatic malignant tumors in 17 (8.9%), and chronic kidney disease in 16 (8.4%). Of the 200 patients with AKI, 41 (20.6%) underwent surgery and 86 (43.2%) developed sepsis. Upon admission, the median serum creatinine level was 0.70 mg/dL (0.54–0.88 mg/dL), which increased to 1.46 mg/dL (1.18–2.10 mg/dL) when AKI was diagnosed. The median SOFA score was 6.0 (4.0–11.0), and 119 patients (59.5%) survived longer than 90 days.

### Vitamin D Concentration in Healthy Subjects, Critically Ill Patients without AKI, and Patients with AKI

The mean age of healthy subjects, critically ill patients without AKI, and patients with AKI was 56.1±18.7, 55.7±16.3, and 63.7±18.8 years, and the proportion of females was 29.4%, 30.8%, and 26.5%, respectively. No statistical difference was found in age or gender among the three subgroups (*p*>0.05). The SOFA scores of critically ill patients without AKI were 7.6±1.8 (normally distributed) and those of patients with AKI were 6.0 (4.0–11.0) (non-normally distributed), with no statistical difference (*p*>0.05).

**Figure 1:**
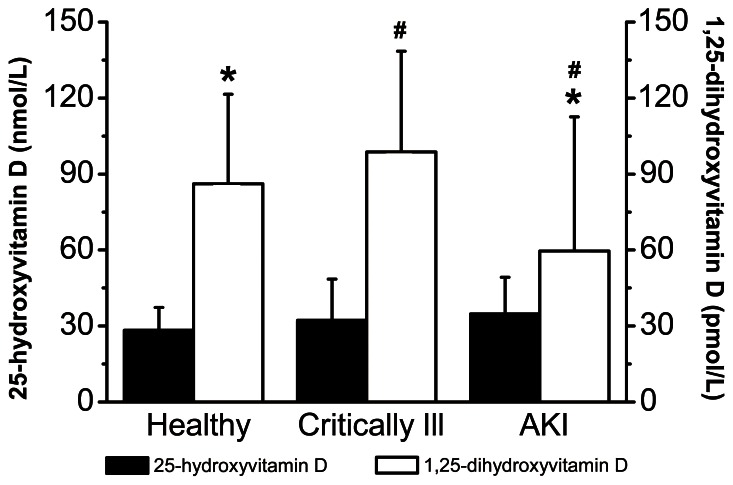
Vitamin D concentrations among healthy subjects, critically ill patients without AKI, and patients with AKI. 25-hydroxyvitamin D: ANOVA, *p* = 0.213. 1,25-dihydroxyvitamin D: ANOVA, *p* = 0.005. *p≤0.05, patients with AKI compared with healthy subjects. #*p*≤0.05, patients with AKI compared with critically ill patients. AKI, acute kidney injury; ANOVA, analysis of variance.

Figure 1 shows the vitamin D concentrations among healthy subjects, critically ill patients without AKI, and patients with AKI. No significant difference in the 25-hydroxyvitamin D level was found among healthy subjects, critically ill patients without AKI, and patients with AKI (28.3±9.0, 32.2±16.2, and 34.7±14.5 nmol/L, respectively; ANOVA, *p* = 0.213). Among patients with AKI, 25-hydroxyvitamin D deficiency (<37.5 nmol/L) was identified in 129 (64.5%) patients, insufficiency (<75 nmol/L) in 69 (34.5%) patients, and sufficiency (≥75 nmol/L) in 2 (1%) patients. Lower concentrations of 1,25-dihydroxyvitamin D were detected in patients with AKI than in healthy subjects and critically ill patients without AKI (59.6±53.0, 86.2±35.3, and 98.8±39.7 pmol/L, respectively). Significant differences were found in the 1,25-dihydroxyvitamin D levels among the three subgroups (ANOVA, *p* = 0.005). The ratio of 1,25-dihydroxyvitamin D to 25-hydroxyvitamin D (×1000) in patients with AKI was 1.81±1.49 and was statistically significant compared with that in healthy subjects (3.61±2.28) and critically ill patients without AKI (3.17±1.23) (ANOVA, *p*<0.001).

### Vitamin D Concentration in Patients with Different RIFLE Stages of AKI

All patients with AKI were classified into three RIFLE subgroups (Risk, Injury, and Failure) according to their changes in serum creatinine levels within 1 week. The sample size of each subgroup (Risk, Injury, and Failure) was 91 (45.5%), 50 (25.0%), and 59 (29.5%) patients, respectively. Comparison among these three subgroups revealed no significant differences in age, gender, or comorbid conditions, but the percentage of patients with operations was highest in the Risk subgroup and lowest in the Failure subgroup. The lowest serum albumin and total calcium levels and the highest C-reactive protein and phosphate levels were found in patients with AKI. The SOFA score, APACHE II score, and SAPS significantly increased with the severity of AKI (*p* = 0.001, 0.001, and 0.007, respectively). The demographic and clinical characteristics as stratified by the RIFLE stage of AKI are shown in Table 1.

**Figure 2:**
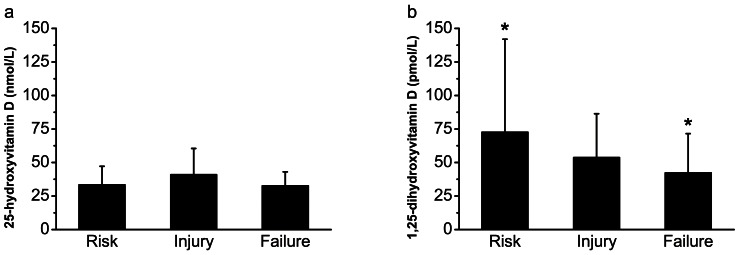
Vitamin D concentrations in 200 patients with AKI stratified by RIFLE stages. (a) 25-hydroxyvitamin D: ANOVA, *p* = 0.083. (b) 1,25-dihydroxyvitamin D: ANOVA, *p* = 0.042. *p≤0.05, patients in Risk stage compared with patients in Failure stage. AKI, acute kidney injury; RIFLE, Risk, Injury, Failure, Loss, and End-stage kidney disease; ANOVA, analysis of variance.

The concentrations of 1,25-dihydroxyvitamin D stratified by the RIFLE criteria were 72.6±69.4, 53.7±32.7, and 42.2±29.3 pmol/L, respectively, with decreasing severity of AKI (ANOVA, *p* = 0.042) (Figure 2b). However, the corresponding concentrations of 25-hydroxyvitamin D were 33.3±13.9, 41.0±19.5, and 32.6±10.5 nmol/L, respectively, showing no correlation with the severity of AKI (ANOVA, *p* = 0.083) (Figure 2a). The ratio of 1,25-dihydroxyvitamin D to 25-hydroxyvitamin D (×1000) was 2.24±1.78 in patients in the Risk subgroup, 1.65±1.36 in the Injury subgroup, and 1.39±0.95 in the Failure subgroup, increasing as the severity of AKI increased (ANOVA, *p* = 0.037).

### Correlation Analysis of Vitamin D with SOFA score and Adjusted Calcium Level

Because the serum calcium concentration is influenced by the serum albumin concentration, we used the adjusted calcium concentration to determine the actual serum calcium concentration. The adjusted calcium concentration was calculated as follows:

adjusted calcium, mmol/L  =  serum total calcium, mmol/L +0.2×(4 – serum albumin, g/dL)

**Figure 3:**
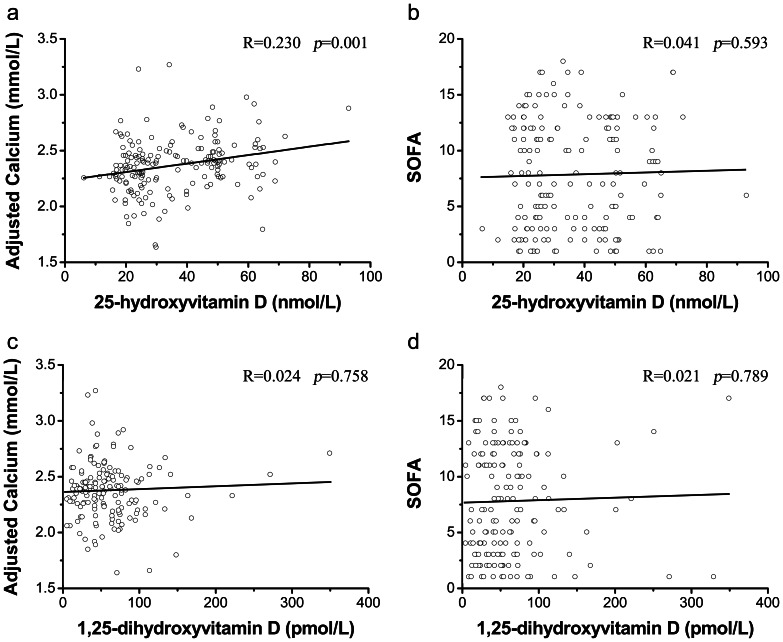
Correlations analysis: vitamin D with adjusted calcium and SOFA score in 200 patients with AKI. (a) Correlation between 25-hydroxyvitamin D and adjusted calcium levels, R = 0.230, adjusted r^2^ = 0.054, *p* = 0.001. (b) Correlation between 25-hydroxyvitamin D level and SOFA score, R = 0.041, adjusted r^2^ = –0.004, *p* = 0.593. (c) Correlation between 1,25-dihydroxyvitamin D and adjusted calcium levels, R = 0.024, adjusted r^2^ = -0.006, *p* = 0.758. (d) Correlation between 1,25-dihydroxyvitamin D level and SOFA score, R = 0.021, adjusted r^2^ = 0.006, *p* = 0.789. AKI, acute kidney injury; SOFA, Sequential Organ Failure Assessment.

The correlation analysis indicated that the 25-hydroxyvitamin D concentration had a statistically significant positive correlation with the adjusted calcium concentration (r = 0.230, adjusted r^2^  = 0.054, *p* = 0.001) (Figure 3a), but that it had no correlation with the SOFA score (Figure 3b). The analysis also showed that the 1,25-dihydroxyvitamin D concentration was not significantly correlated with either the adjusted calcium concentration or the SOFA score (Figure 3c, d). We also found no correlation of the vitamin D concentration with the body mass index or serum levels of cholesterol, albumin, phosphate, or C-reactive protein (data not shown).

### Mortality Predictability of Vitamin D during a 90-Day Period

We further divided the 200 patients with AKI into two subgroups according to the median serum concentration of 25-hydroxyvitamin D: the low 25-hydroxyvitamin D level group (<30.5 nmol/L) and the high 25-hydroxyvitamin D level group (>30.5 nmol/L). We divided these patients into two additional subgroups according to the median serum concentration of 1,25-dihydroxyvitamin D: the low 1,25-dihydroxyvitamin D level group (<44.3 pmol/L) and the high 1,25-dihydroxyvitamin D level group (>44.3 pmol/L).

**Figure 4:**
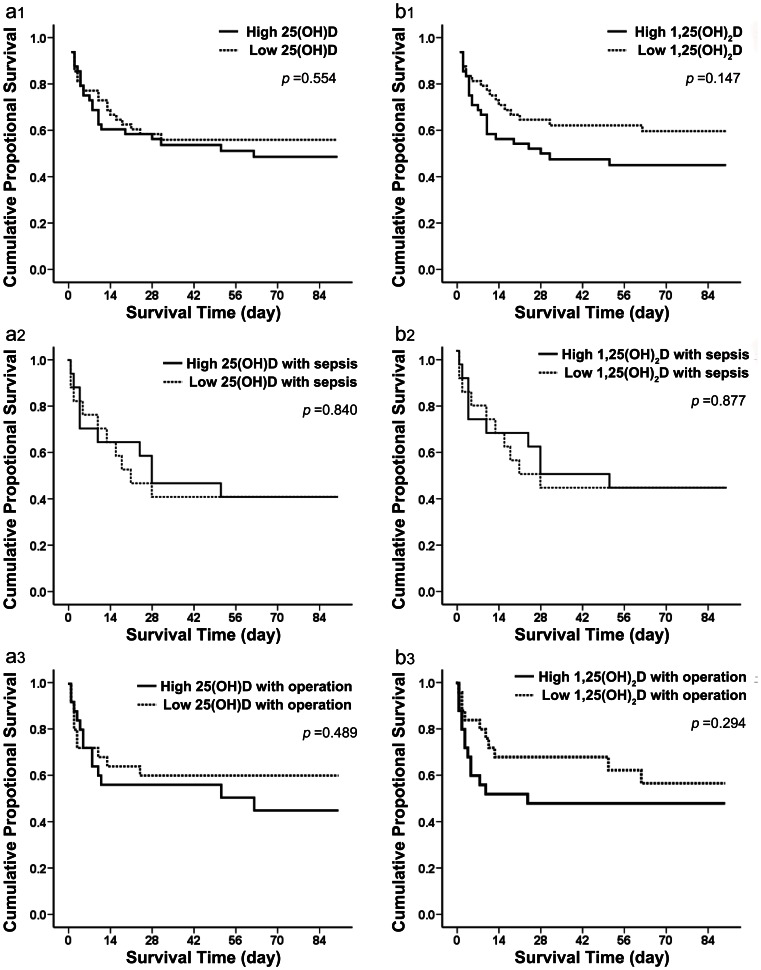
Ninety-day survival rate in patients with AKI with different vitamin D concentrations. (a1) Failure plots for probability of survival at 90 days between high and low 25-hydroxyvitamin D level subgroups. No significant difference between the two subgroups was found (log rank, *p* = 0.554). (b1) Failure plots for probability of survival at 90 days between high and low 1,25-dihydroxyvitamin D level subgroups. No significant difference between the two subgroups was found (log rank, *p* = 0.147). (a2) Failure plots for probability of survival at 90 days between high and low 25-dihydroxyvitamin D level subgroups including patients with sepsis. No significant difference between the two subgroups was found (log rank, *p* = 0.840). (b2) Failure plots for probability of survival at 90 days between high and low 1,25-dihydroxyvitamin D level subgroups including patients with sepsis. No significant difference between the two subgroups was found (log rank, *p* = 0.877). (a3) Failure plots for probability of survival at 90 days between high and low 25-dihydroxyvitamin D level subgroups including patients with operations. No significant difference between the two subgroups was found (log rank, *p* = 0.489). (b3) Failure plots for probability of survival at 90 days between high and low 1,25-dihydroxyvitamin D level subgroups including patients with operations. No significant difference between the two subgroups was found (log rank, *p* = 0.294).

Among patients in the high 25-hydroxyvitamin D level group (mean, 45.6±12.7 nmol/L), 22 died (45.8%) compared with 23 (47.9%) in the low 25-hydroxyvitamin D level group (mean, 23.9±5.1 nmol/L). The survival curve showed no significant difference between those two 25-hydroxyvitamin D groups according to the Kaplan-Meier plot (*p* = 0.554 by the log rank test) (Figure 4a1). In addition, 21 patients (43.8%) in the high 1,25-dihydroxyvitamin D level group (mean, 87.9±58.0 pmol/L) died compared with 24 (50.0%) in the low 1,25-dihydroxyvitamin D level group (mean, 28.2±14.1 pmol/L). The survival curve also showed no significant difference between those two 1,25-dihydroxyvitamin D groups according to the Kaplan-Meier plot (*p* = 0.147 by the log rank test) (Figure 4b1).

Among all patients with AKI, we then selected those who developed sepsis and those who underwent surgical operations and divided them into two subgroups according to the median 25-hydroxyvitamin D and 1,25-dihydroxyvitamin D concentrations, respectively. Among the septic patients, the survival curve showed no significant difference between the high and low 25-hydroxyvitamin D level groups (sepsis group: *p* = 0.840 by the log rank test) (Figure 4a2) or between the high and low 1,25-dihydroxyvitamin D level groups (sepsis group: *p* = 0.877 by the log rank test) (Figure 4b2) according to the Kaplan-Meier plot. Among the surgical patients, the survival curve showed no significant difference between the high and low 25-hydroxyvitamin D level groups (sepsis group: *p* = 0.489 by the log rank test) (Figure 4a3) or between the high and low 1,25-dihydroxyvitamin D level groups (operation group: *p* = 0.294 by the log rank test) (Figure 4b3) according to the Kaplan-Meier plot.

Finally, we found no association between the ratio of 1,25-dihydroxyvitamin D to 25-hydroxyvitamin D and all-cause mortality in patients with AKI (data not shown).

### Mortality Predictability of VDR Polymorphism during a 90-Day Period

VDR is synthesized by the VDR gene, which is located on chromosome 12 and contains 9 exons. DNA sequence variations referred to as “polymorphisms” of the VDR, which occur frequently in the general population, may affect the degree of gene expression, mRNA stability, and protein translation efficiency, thus affecting the levels and affinity of the VDR protein and the resultant action of the vitamin D–VDR complex.

We measured two classic VDR polymorphisms, *Fok*I and *Bsm*I, to determine whether they are linked to early death in patients with AKI and to use them as covariates for adjustment in multivariate analyses. The *Fok*I *f* allele is known to encode a protein that is three amino acids shorter and has higher transcriptional activity than the wild-type (*FF*) protein [14]. The serum level of 1,25-dihydroxyvitamin D is higher in individuals who are homozygous for the *Bsm*I *B* allele (*BB*) than in individuals who are heterozygous or homozygous for the *b* allele [19].

**Figure 5:**
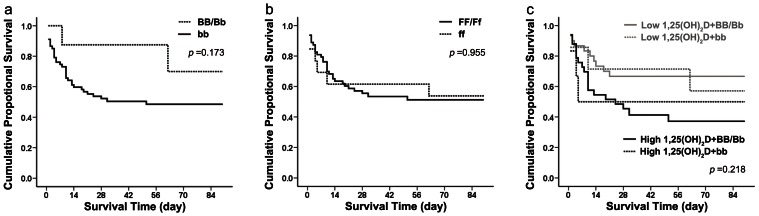
Ninety-day survival rate in patients with AKI with different genotypes. (a) Failure plots for probability of survival at 90 days between the *BB/Bb* and *bb* genotypes. No significant difference between the two subgroups was found (log rank, *p* = 0.173). (b) Failure plots for probability of survival at 90 days between the *FF/Ff* and *ff* genotypes. No significant difference between the two subgroups was found (log rank, *p* = 0.955). (c) Failure plots for probability of survival at 90 days among patients with the *FF/Ff* genotype with high 1,25-dihydroxyvitamin D levels, the *FF/Ff* genotype with low 1,25-dihydroxyvitamin D levels, the *ff* genotype with high 1,25-dihydroxyvitamin D levels, and the *ff* genotype with low 1,25-dihydroxyvitamin D levels. No significant difference among the four subgroups was found (log rank, *p* = 0.218).

Table 3 shows the genotype and allele distributions for the VDR gene *Fok*I and *Bsm*I polymorphisms in patients with AKI. The distribution of the observed *Fok*I and *Bsm*I polymorphisms and genotype frequencies fit the Hardy–Weinberg predictions, suggesting that the alleles were in equilibrium (*p*>0.05). Among patients with AKI, 2 (1.3%) had the *BB* genotype, 14 (9.3%) had the *Bb* genotype, and 134 (89.3%) had the *bb* genotype, while 48 (31.6%) had the *FF* genotype, 78 (51.3%) had the *Ff* genotype, and 26 (17.1%) had the *ff* genotype. These frequencies were similar to those in the general population as reported in other Chinese studies. We further divided patients with AKI into two subgroups: the *BB/Bb* versus *bb* genotype according to the *Bsm*I polymorphism and the *FF/Ff* versus *ff* genotype according to the *Fok*I polymorphism. Based on the Kaplan-Meier plot (Figure 5), the survival curves of patients with the *BB/Bb* or *FF/Ff* genotype showed no significant difference compared with the survival curves of patients with the *bb* or *ff* genotype. At 90 days after patient enrollment, the mortality rate was not significantly higher in the group with the *ff* genotype (*p* = 0.955 by the log rank test) (Figure 5a) or the *bb* genotype (*p* = 0.173 by the log rank test) (Figure 5b). Twenty-five percent of patients with the *BB/Bb* genotype died compared with 50.7% of patients with the *bb* genotype, and 47.6% of patients with the *FF/Ff* genotype died compared with 46.2% of patients with the *ff* genotype. We then classified patients with AKI by both the 1,25-dihydroxyvitamin D level and the *Fok*I polymorphism status, but the survival curves still showed no significant difference among the four groups (*p* = 0.218 by the log rank test) (Figure 5c). Furthermore, no relationship was found between VDR polymorphisms and serum vitamin D levels (data not shown).

**Table 3:** Distributions of the *Fok*I and *Bsm*I VDR gene polymorphisms in patients with AKI.

*Bsm*I polymorphism	n (%)	*Fok*I polymorphism	n (%)
Genotype-wise comparison	n = 150*	Genotype-wise comparison	n = 152
*BB*	2 (1.33)	*FF*	48 (31.58)
*Bb*	14 (9.33)	*Ff*	78 (51.32)
*bb*	134 (89.33)	*ff*	26 (17.11)
*BB* and *Bb*	16 (10.67)	*FF* and *Ff*	126 (82.89)
Allele-wise comparison		Allele-wise comparison	
*B*	18 (6.00)	*F*	174 (57.24)
*b*	282 (94.00)	*f*	130 (42.76)

**Abbreviations:** VDR, vitamin D receptor; AKI, acute kidney disease.

### Multivariate Analysis of Selected Possible Predictors for Mortality

Cox proportional hazards regression was used to identify independent predictors of mortality in patients with AKI. Covariates including age, gender, SOFA score, and VDR polymorphisms including *Bsm*I and *Fok*I were used for stepwise adjustment. The results of multivariate Cox regression analysis for the vitamin D level and other selected possible predictors of mortality in patients with AKI (Table 4) showed that regardless of adjustment for age, gender, severity of illness (SOFA score), and VDR polymorphisms, neither the 25-hydroxyvitamin D nor the 1,25-dihydroxyvitamin D level was an independent predictor of 90-day overall mortality in patients with AKI (*p*>0.05).

**Table 4:** Multivariate Cox regression analysis for vitamin D and other selected possible predictors of mortality in patients with AKI.

	25(OH)Dnmol/L		1, 25(OH)_2_Dpmol/L		*Bsm*I		*Fok*I		Age		Gender		SOFA score	
	HR	*p*	HR	*p*	HR	*p*	HR	*p*	HR	*p*	HR	*p*	HR	*p*
Model 1	1.007	0.522	1.003	0.326	/	/	/	/	/	/	/	/	/	/
Model 2	1.006	0.571	1.003	0.404	0.409	0.224	1.172	0.739	/	/	/	/	/	/
Model 3	1.013	0.799	1.002	0.514	0.360	0.168	1.131	0.799	0.987	0.172	1.148	0.737	/	/
Model 4	1.007	0.593	1.004	0.136	0.759	0.721	0.639	0.360	0.998	0.843	0.571	0.220	1.314	<0.001

**Note:** The multivariate analysis sequentially adjusted the models for covariates as follows: Model 1, unadjusted; Model 2, adjusted for VDR polymorphisms; Model 3, adjusted for age, gender, and VDR polymorphisms; Model 4, adjusted for age, gender, SOFA score, and VDR polymorphisms.

**Abbreviations:** 25(OH)D, 25-hydroxyvitamin D; 1,25(OH)_2_D, 1,25-dihydroxyvitamin D; SOFA, Sequential Organ Failure Assessment.

## Discussion

The goal of the present study was to measure the vitamin D levels in patients with AKI and determine whether the vitamin D level at the time of AKI diagnosis is associated with 90-day overall mortality. Our observational study showed that patients with AKI manifested a marked decrease in the serum 25-hydroxyvitamin D and 1,25-dihydroxyvitamin D levels at the time of AKI diagnosis and that the degree of 1,25-dihydroxyvitamin D deficiency increased as the severity of AKI increased. No association between either the 25-hydroxyvitamin D or 1,25-dihydroxyvitamin D level at the time of AKI diagnosis and 90-day all-cause mortality was found in patients with AKI.

Serum 25-hydroxyvitamin D deficiency in critically ill patients before or at ICU admission has been described in several earlier observational studies [8]–[13]. According to Braun et al. [14], the preadmission serum 25-hydroxyvitamin D level was measured at 67.7 nmol/L in patients with AKI. However, the 25-hydroxyvitamin D level at the time of AKI diagnosis has been rarely reported. In the present study, the mean serum 25-hydroxyvitamin D concentration at the time of AKI diagnosis was 34.75 nmol/L in patients with AKI, which is defined as a deficiency according to the Western classification [12], [13], [20]. However, the serum 25-hydroxyvitamin level in healthy subjects was as low as that of both critically ill patients and patients with AKI. It seems that 25-hydroxyvitamin D deficiency was prevalent in our study population. Similarly, a cohort study conducted by another tertiary hospital in Shanghai involving 452 healthy adults demonstrated a serum 25-hydroxyvitamin D level of 42.2 nmol/L in women aged 50 to 70 years [21]. Different races, latitudes, climates, diets, and clothing might account for the lower 25-hydroxyvitamin D levels in Shanghai compared with those in the Western general population. A large, multicenter, epidemiological survey on vitamin D levels is needed in China to establish new 25-hydroxyvitamin D classification criteria suitable for the Chinese population. In addition, no significant difference in the 25-hydroxyvitamin D level was found among patients with different RIFLE stages in the present study. The lack of adjustment for various lengths of hospital stays before AKI diagnosis, and different methods of nutritional support may have partly accounted for the lack of statistical significance.

In contrast to previous studies [8]–[13], [20], we focused on the serum 25-hydroxyvitamin D level at the time of AKI diagnosis in patients with AKI. We observed no evidence of a relationship between 25-hydroxyvitamin D deficiency and overall mortality, even in analyses restricted to patients with sepsis or to patients in whom operations were performed within 1 week according to the Kaplan-Meier method or Cox proportional hazards regression analysis, regardless of adjustment. The prevalent deficiency of 25-hydroxyvitamin D in our study population may be a reason for the lack of statistical significance. Because most of the patients with AKI who were enrolled in our study had 25-hydroxyvitamin D insufficiency, the disparity in the mean 25-hydroxyvitamin D level between the high and low level groups was so small that it may not have shown any difference in mortality. Actually, according to previous observational studies, whether the observed association between serum 25-hydroxyvitamin D deficiency prior to hospital admission or around the time of ICU admission and increased all-cause mortality in critically ill patients is the cause of the increased mortality or a co-factor of disease severity remains controversial [13]. The different cohort characteristics including race, comorbid conditions, and sepsis morbidity may have also contributed to the different results. Furthermore, Braun et al. found that serum 25-hydroxyvitamin D deficiency prior to hospital admission is a significant predictor of AKI [20].

25-Hydroxyvitamin D is converted to active 1,25-hydroxyvitamin D by 1α-hydroxylase in the renal proximal tubules. This infers that the potential sudden loss or malfunction of 1α-hydroxylase in the kidney would reduce the formation of 1,25-dihydroxyvitamin D from 25-hydroxyvitamin in patients with AKI, similar to the mechanism of hypovitaminosis D in patients with chronic kidney disease [22]. Thus, we measured the serum 1,25-dihydroxyvitamin D level as well. Our study demonstrated that 1,25-dihydroxyvitamin D deficiency obviously occurred in patients with AKI compared with normal subjects and critically ill patients without AKI, and the degree of deficiency increased as the severity of AKI increased. This implies that 1,25-dihydroxyvitamin D is a marker that can reflect kidney injury.

Although 1,25-dihydroxyvitamin D deficiency occurred in patients with AKI compared with critically ill patients without AKI and healthy subjects, we still observed no evidence of a relationship between 1,25-dihydroxyvitamin D deficiency and increased mortality in patients with AKI. This finding was consistent even in analyses restricted to patients with sepsis or to patients who underwent operations within 1 week according to Kaplan-Meier survival analysis or Cox proportional hazards regression analysis, regardless of adjustment.

The prevalent deficiency of 25-hydroxyvitamin D in patients with AKI may have also been one of the reasons for the lack of statistical significance regarding the mortality of the two 1,25-dihydroxyvitamin D groups. The 1,25-dihydroxyvitamin D level in most of the study population was low because the prohormone, circulating 25-hydroxyvitamin D, was insufficient; this may have led to the lack of a difference in mortality between the two groups. The autocrine or paracrine function of the extrarenal vitamin D system in some target tissues, such as immune cells or the colon, might help to quickly complement the adverse effect of the low circulating 1,25-dihydroxyvitamin D level caused by AKI and maintain the pleiotropic actions of vitamin D [23]. Measurement of extrarenal 1,25-dihydroxyvitamin D or 1α-hydroxylase in various local tissues is needed to elucidate the potential mechanism. Despite adjustment for multiple potential confounders, residual confounding caused by unmeasured or unadjusted variables (e.g., parathyroid hormone or fibroblast growth factor 23) may have contributed to the observed outcomes.

The effect of active vitamin D on the human body can be potentially influenced by activation of the VDR. The VDR acts as a ligand-activated transcription factor that alters the transcription rates of target genes responsible for biological functions, such as induction of cell differentiation, inhibition of cell growth, immunomodulation, and control of other hormonal systems [1]. It is generally accepted that VDR polymorphisms are linked to increased long-term mortality in the general population as evidenced by their high incidence in several chronic complications, such as coronary artery disease, osteoporosis, autoimmune disorders, diabetes, and cancer [16]. To the best of our knowledge, the association between VDR polymorphisms and short-term mortality remains unclear. To determine whether VDR polymorphisms are linked to mortality in patients with AKI and use them as covariates for adjustment in multivariate analyses, we measured the two classic VDR polymorphisms, *Fok*I and *Bsm*I. *Fok*I polymorphism results in a VDR molecule that is three amino acids shorter and that has higher biological activity than the wild-type protein. In addition, *Bsm*I polymorphism may affect mRNA stability [21]. However, we also observed no evidence of a relationship between *Fok*I or *Bsm*I polymorphisms and early overall death in patients with AKI, even in analyses restricted to patients with sepsis or to patients who underwent operations within 1 week. Importantly, regardless of adjustment for *Fok*I and *Bsm*I polymorphisms, neither the 25-hydroxyvitamin D nor the 1,25-dihydroxyvitamin D level was an independent predictor of 90-day overall mortality in patients with AKI. Because we detected only two polymorphisms among the known single nucleotide polymorphisms of the VDR, it remains unclear whether other VDR polymorphisms are associated with overall mortality and should be adjusted for.

We are aware of the limitations of our small, single-center, observational cohort study. Despite the exclusion of patients with AKI definitively or possibly caused by acute glomerulonephritis, acute interstitial nephritis, renal vasculitis, or postrenal disease, the causes of AKI in our study are very complex and multiplex, potentially confounding one another. Critically ill patients also had multiple other reasons for their mortality, which should be adjusted for. However, the sample size of our study was not large enough for us to perform a subgroup analysis of causality or adjust for additional influencing variables. Moreover, some factors that can alter vitamin D levels, such as smoking status and lack of sun exposure, cannot be adjusted for. This is a potentially important reason for the lack of statistical significance. Larger cohorts are needed in future studies. Serial measurements of the 25-hydroxyvitamin D or 1,25-dihydroxyvitamin D level are also suggested to determine whether these levels are normal before the development of AKI, how the vitamin D status changes with the progression of AKI, and whether the vitamin D status at different stages is associated with prognosis.
